# Inter‐Brain Neural Couplings During Table Tennis Doubles

**DOI:** 10.1002/pchj.70090

**Published:** 2026-04-21

**Authors:** Weixia Zhang, Hangguo Yang, Mengbi Yang, Tiantian Yin, Huiying Liu, Shijie Lin, Shubin Si

**Affiliations:** ^1^ Department of Physical Education Northwestern Polytechnical University Xi'an China; ^2^ School of Public Policy and Administration Northwestern Polytechnical University Xi'an China; ^3^ Department of Industrial Engineering Northwestern Polytechnical University Xi'an China

**Keywords:** hyperscanning, interpersonal synchronization, joint anticipation, neural basis, table tennis doubles

## Abstract

fNIRS‐based hyperscanning showed increased inter brain synchronization in DLPFC and pars triangularis during joint anticipation versus rest in table tennis doubles, suggesting a neural basis for efficient player cooperation.

Cooperation is a cornerstone of social interaction, and many cooperative behaviors rely on synchronized anticipation. In racket sports, the ability to anticipate an opponent's action is a fundamental perceptual‐cognitive skill essential for overcoming severe temporal constraints and successfully intercepting a ball (Loffing and Cañal‐Bruland 2017). While many studies have explored the neural basis of individual anticipation, for instance, by having participants judge the direction (Runswick et al. 2020) or landing point of a ball (Zhao et al. 2018), cooperative tasks like table tennis doubles introduce an additional layer of complexity.

In team sports like table tennis doubles, performance depends not only on individual anticipation but also on joint anticipation. Joint anticipation is the coordinated capacity of a dyad to predict future events and synchronize their actions under conditions of incomplete information and time pressure. This ability to make joint inference under uncertainty is a fundamental driver of performance and epitomizes the cooperative demands of table tennis doubles.

Hyperscanning, the simultaneous recording of brain activity from two or more individuals, offers an ideal technique for examining the neural basis of such cooperative behaviors. Functional Near‐Infrared Spectroscopy (fNIRS) hyperscanning studies have consistently reported higher values of inter‐brain synchronization (IBS) in cooperation tasks across several brain regions, including the dorsolateral prefrontal cortex (DLPFC), inferior frontal gyrus (IFG), and temporoparietal regions (Czeszumski et al. 2022). However, these prior studies have largely focused on non‐anticipatory tasks, such as problem‐solving, cooperative singing or tangram puzzles. In contrast, table tennis doubles require players to respond under uncertainty and rapid temporal constraints. Thus, the neural basis of this distinct form of cooperation (joint anticipation under uncertainty) remains unexplored. Given the critical role of anticipation and dyadic cooperation in table tennis doubles, this study aims to reveal the neural correlates of joint anticipation using fNIRS hyperscanning under conditions specific to this sport.

Sixty‐two unique table tennis players were recruited (49 males, 13 females; *M*_age_ = 21.41, SD = 2.08). All participants held a national athlete certification of Level II or above and had substantial experience in competitive doubles play. Participants could complete the experiment multiple times, each time paired with a different partner. This yielded 48 unique dyads (9 mixed‐gender, 31 male–male, 8 female–female) for the analysis. To engage anticipatory processes, visual stimuli were presented from a first‐person perspective corresponding to the opposite side of the table. Stimuli consisted of frames extracted from pre‐recorded video footage, selected to depict the moment before ball‐racket contact. The exact time point for each stimuli was determined through consensus between two professional players, who identified frames where the judgment of the ball's trajectory was ambiguous without communication between partners (see upper part of Figure 1A for an example).

**FIGURE 1:**
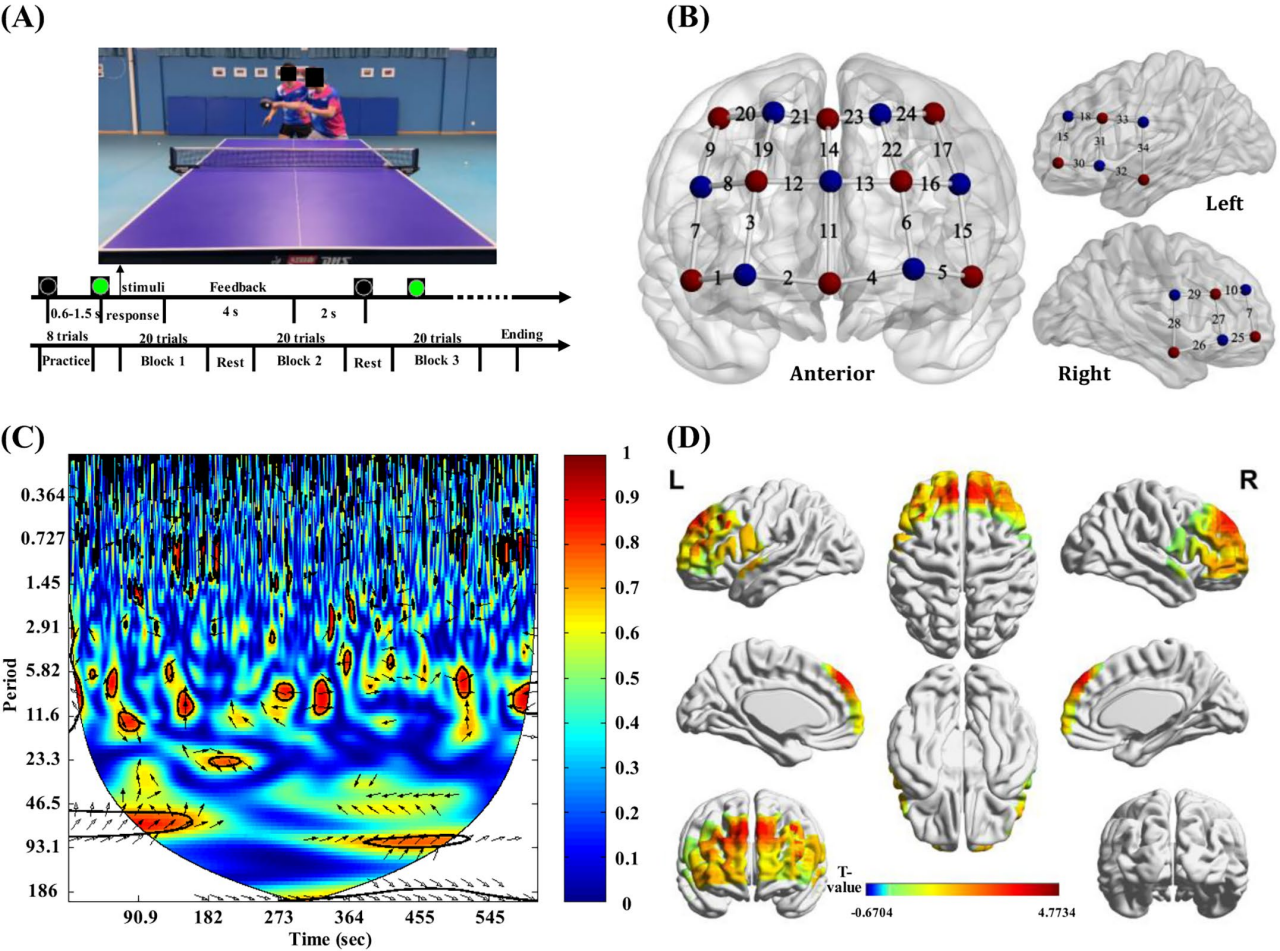
(A) Stimuli used in the study. The faces of the individuals in Figure A have been occluded considering their portrait rights. Trial structure (upper time flow) and experiment procedure (lower time flow); (B) Cap configuration. Red points are emitters, blue points are detectors, and numbers indicate channels; (C) WTC from CH17 for the 12th pair of participants; (D) *t* value of coherence increase for group analysis.

During the experiment, participants were required to judge the ball's landing direction by pressing keys (Player1: Z/left, X/right; Player 2: N/left, M/right, see lower part of Figure 1A). Behavioral performance was computed based on three dimensions: the difference in reaction times (RT) (+1 if the absolute RT difference was less than 1/8 of their combined RTs, −1 if greater); the consistency of their keypress responses (+1 for congruent responses, −1 for incongruent responses); and the accuracy of their anticipation (+1 if both responses were correct, otherwise −1). This scoring method reflected core demands of real‐world table tennis doubles, emphasizing speed, synchronization, and accuracy.

Neural signals from both players were collected using a 34‐channel fNIRS system (NirSmartII‐500Pro, HuiChuang, Beijing) (see Figure 1B for cap configuration). After preprocessing and quality assessment, the fNIRS signals were converted to optical density (OD) changes and subsequently transformed into concentrations changes of oxygenated hemoglobin (HbO) and deoxygenated hemoglobin (HbR). Subsequent analyses focused on the HbO signal due to its superior signal‐to‐noise ratio. To assess the IBS, we applied Wavelet Transform Coherence (WTC), a method widely used in fNIRS hyperscanning studies.

The frequency of interest (FOI) for the WTC analysis was set to 0.08–0.15 Hz. This band encompasses the Mayer wave (~0.1 Hz), which is strongly associated with task‐evoked neurovascular coupling and is less contaminated from higher‐frequency physiological noises. Moreover, this band has been successfully employed in prior investigations of cooperative behaviors (Wang et al. 2019). We also conducted an analysis using the broader 0.08–0.3 Hz band as in Cui et al. (2012); however, no significant IBS was found, confirming the appropriateness of our band selection. WTC analysis generated a 2‐D coherence map (Figure 1C).

Coherence increase was defined as the average coherence value in the task minus coherence value in the rest. These difference scores for each channel were then subjected to one‐sample *t*‐tests at the group level, with results presented in Figure 1D. *p* values were corrected for multiple comparisons using the false discovery rate (FDR). Significant increases in IBS were found in channel 8 (*t* = 3.02, *p* < 0.05, *d* = 0.44), channel 14 (*t* = 3.76, *p* < 0.01, *d* = 0.55), channel 17 (*t* = 4.77, *p* < 0.001, *d* = 0.70) and channel 15 (*t* = 2.95, *p* < 0.05, *d* = 0.4). Channels 8, 14, 17 overlay the bilateral DLPFC, while channel 15 is located in the pars triangularis of Broca's Area, part of the IFG. These brain regions have consistently been reported in hyperscanning studies of cooperation (Cui et al. 2012; Czeszumski et al. 2022).

To directly link our findings to table tennis expertise and task specificity, both control group and control task were included. The results showed that the above pattern were not observed, suggesting that observed enhancement of IBS is experience‐dependent (differentiating athletes from amateurs) and cognitively specific (linked to joint anticipation rather than general cooperation). Full details of these control analyses are provided in the Supporting Information. It should be noted that the inclusion of these control conditions was solely to validate the specificity and robustness of our primary findings. These additions did not alter the original experimental design, core task, data acquisition protocol, or analytical pipeline for the main experiment. The original design was retained because it was specifically developed to elicit the cognitive processes central to our research question: joint anticipation under uncertainty.

The increased IBS suggest that our experimental task effectively elicits cooperative neural processes in table tennis doubles. The involvement of the DLPFC is related to inferring the intentions of others (Rilling et al. 2004), while activation in the left pars triangularis aligns with the efficient integration of information under uncertainty (Toelch et al. 2014), the increased IBS in our study may reflect collaborative information integration based on inferring intentions of opponents in table tennis doubles.

Based on the significant increases in IBS, we further assessed its relationship with the sum scores and each behavioral dimension through Pearson correlation. Results showed a significant positive correlation only between IBS in channel 17 and the total score (*r* = 0.39, *p* < 0.01). This result suggests that IBS in this region may support the integrated process of coordinating speed, alignment, and judgment under uncertainty, rather than being specifically tied to any single behavioral component.

In summary, we report the first fNIRS‐based investigation of inter‐brain coupling between table tennis doubles during joint anticipation. Increased synchronization in the DLPFC and the pars triangularis during anticipatory tasks suggests that these brain regions may underpin joint anticipation in table tennis doubles. Moreover, heightened synchronization in the DLPFC is a potential neural indicator of efficiency in interactive sports contexts.

## Funding

This work was supported by the Shaanxi Provincial Natural Science Foundation (2023JCQ0258), Shaanxi Province “14th Five‐Year” Education Science Planning Project (SGH24Q473) and Education and Teaching Reform Research Project of Northwestern Polytechnical University (2025JGZ57).

## Ethics Statement

The procedure received ethical approval from the ethic committee of Northwestern Polytechnical University, and that written informed consent was obtained from participants.

## Conflicts of Interest

The authors declare no conflicts of interest.

## Supporting information


**Data S1:** Supporting Information.

## Data Availability

The data that support the findings of this study are available on request from the corresponding author. The data are not publicly available due to privacy or ethical restrictions.
