# Supplementary material for: Inter‐Brain Neural Couplings During Table Tennis Doubles
Source: Psych J. 2026 Apr 21;15(2):e70090. doi: 10.1002/pchj.70090 (PMC13099260; doi:10.1002/pchj.70090)
Supplement: Supplementary file 1 — Data S1: Supporting Information. [file PCHJ-15-e70090-s001.docx]

To directly link our findings to table tennis, both control groups and control task were included. The control group consisted of 48 dyads of table tennis amateurs without formal professional training (Mage = 19.67, SD = 1.46; 14 mixed-gender, 29 mal-male, 5 female-female). Demographic variables, including age, gender, and dyad type, were matched with the athlete group (*ps* < .05). **Due to a data collection error, data from the general cooperation task of were missing for one amateur dyad. In addition, four athlete dyads cannot be reached for follow-up testing because of personal scheduling conflicts, resulting in missing general cooperation task data for these participants. Following are results in details.**

**1. Results of table tennis task in amateurs**

During the table tennis anticipation task, amateurs exhibited significantly increased IBS at CH32 (t = 3.39, *p* = 0.049, d = 0.49). The corresponding t-map and statistical results are shown in Figure 1 and Table 1, respectively. Critically, this pattern of IBS observed in amateurs did not replicate the findings previously obtained from expert athletes. This new finding suggests that our previous results could be specifically linked to expert-level table tennis performance, rather than reflecting a general feature of dyadic task engagement.


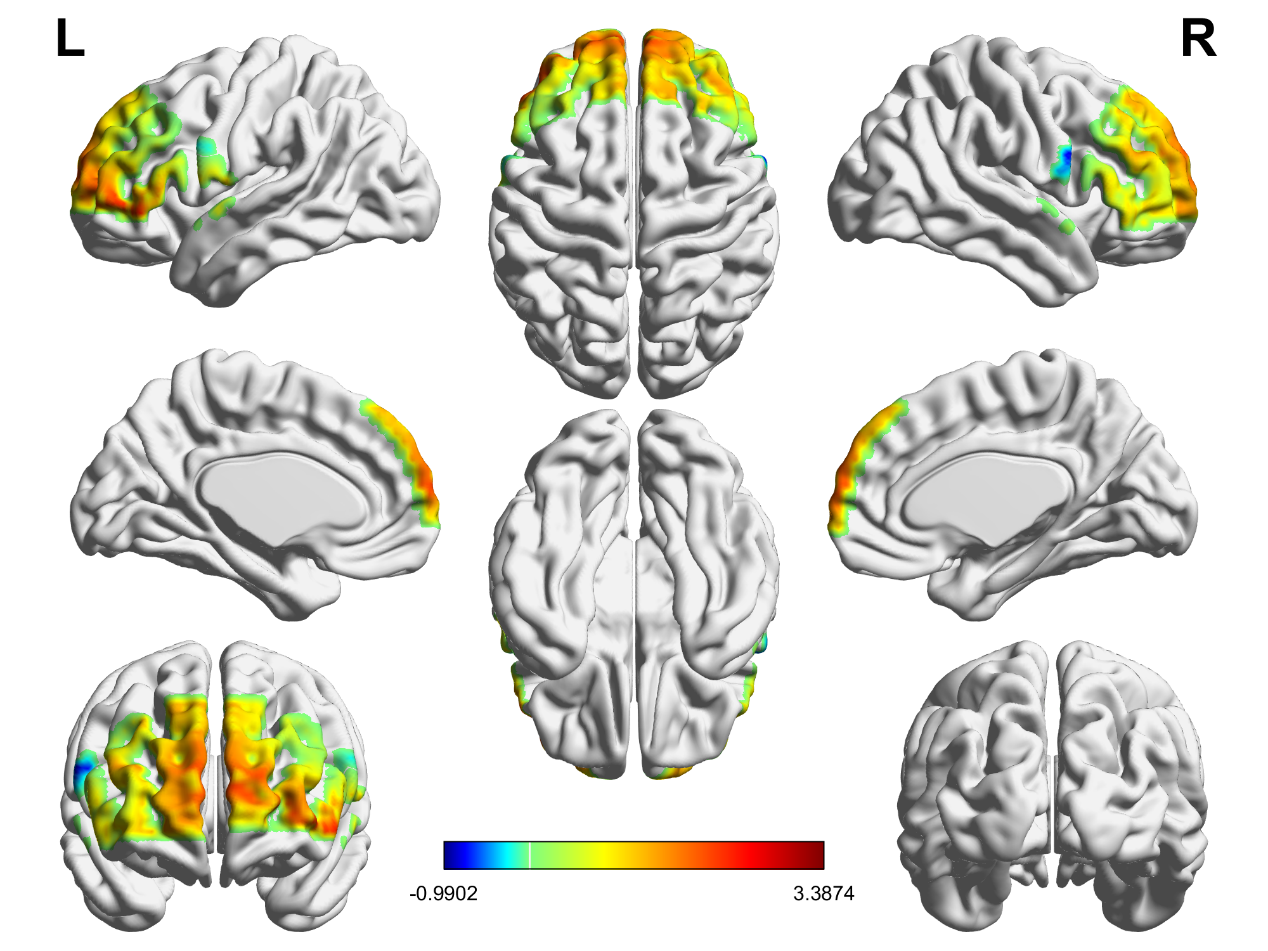


**Figure 1.** T map during table tennis task in amateurs.

**Table 1.** Results of T test during table tennis task in amateurs

| Channel | Corrected_P_Value | T_Value | Cohen_D |
| --- | --- | --- | --- |
| 1 | 0.59 | 1.00 | 0.14 |
| 2 | 0.40 | 1.48 | 0.21 |
| 3 | 0.40 | 1.41 | 0.20 |
| 4 | 0.69 | 0.60 | 0.09 |
| 5 | 0.28 | 2.01 | 0.29 |
| 6 | 0.07 | 3.00 | 0.43 |
| 7 | 0.94 | 0.16 | 0.02 |
| 8 | 0.69 | 0.71 | 0.10 |
| 9 | 0.84 | 0.34 | 0.05 |
| 10 | 0.40 | 1.55 | 0.22 |
| 11 | 0.26 | 2.14 | 0.31 |
| 12 | 0.17 | 2.53 | 0.36 |
| 13 | 0.29 | 1.81 | 0.26 |
| 14 | 0.50 | 1.25 | 0.18 |
| 15 | 0.68 | 0.82 | 0.12 |
| 16 | 0.69 | 0.57 | 0.08 |
| 17 | 0.59 | 1.04 | 0.15 |
| 18 | 0.94 | 0.07 | 0.01 |
| 19 | 0.69 | 0.55 | 0.08 |
| 20 | 0.40 | 1.45 | 0.21 |
| 21 | 0.94 | 0.11 | 0.02 |
| 22 | 0.68 | 0.78 | 0.11 |
| 23 | 0.29 | 1.84 | 0.27 |
| 24 | 0.69 | 0.67 | 0.10 |
| 25 | 0.69 | 0.55 | 0.08 |
| 26 | 0.29 | 1.83 | 0.26 |
| 27 | 0.68 | 0.85 | 0.12 |
| 28 | 0.94 | 0.09 | 0.01 |
| 29 | 0.59 | -0.99 | -0.14 |
| 30 | 0.19 | 2.37 | 0.34 |
| 31 | 0.54 | 1.15 | 0.17 |
| **32** | 0.05 * | 3.39 | 0.49 |
| 33 | 0.69 | -0.54 | -0.08 |
| 34 | 0.33 | 1.69 | 0.24 |

Note: “*” denotes *p* < .05 after FDR correction. Significant channels are bolded in the first column.

**2. Results of general cooperation task in athletes**

A control task involving general cooperation, replicated from Cui et al. (2012), was used. Results indicated that, compared to the resting state, the athlete group exhibited significantly enhanced IBS in CH34 during the general cooperative task (t = 3.77, *p* = 0.02, d = 0.58), see Figure 2 and Table 2.


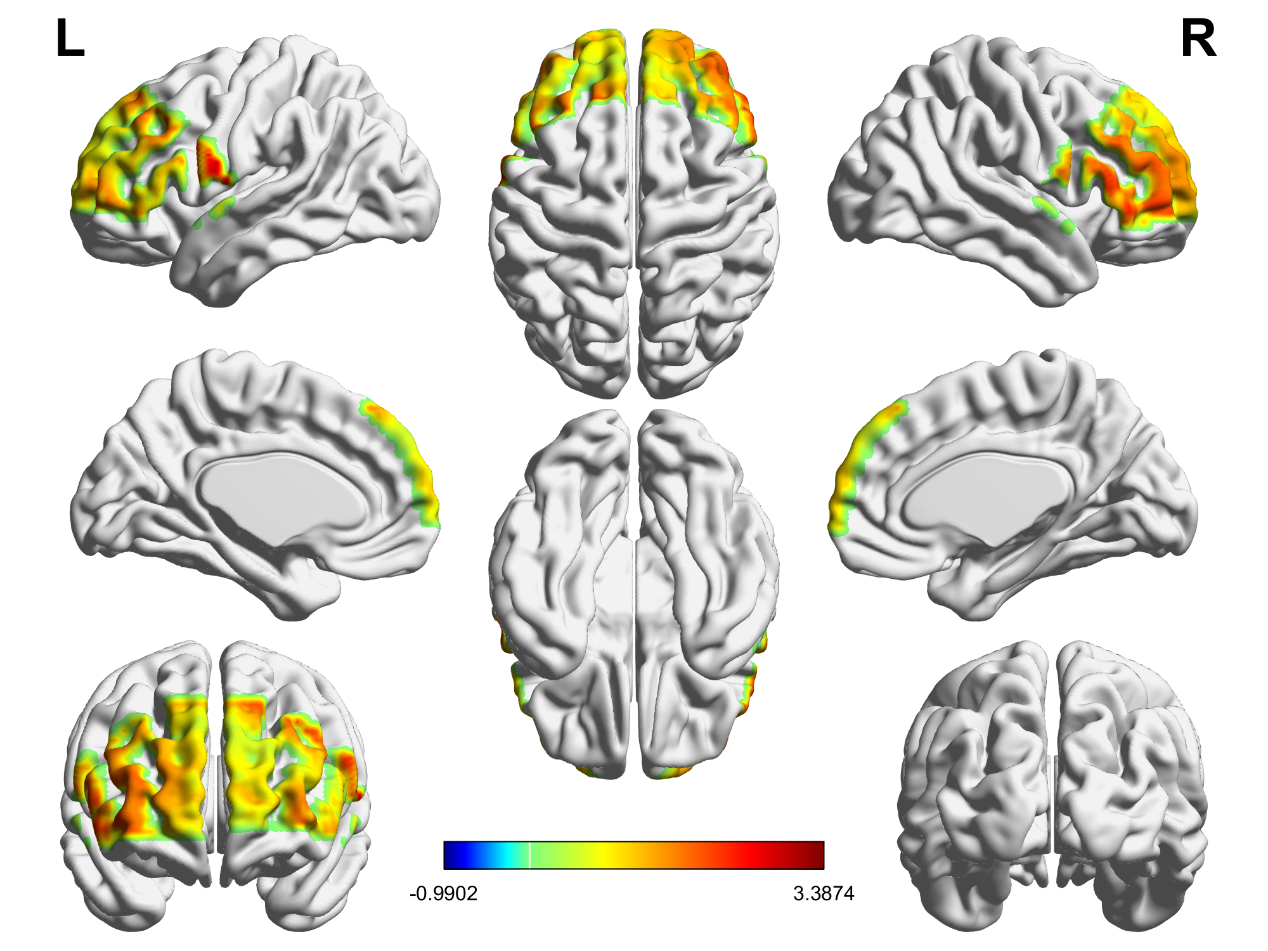


**Figure 2.** T map during general cooperation task in athletes.

**Table 2.** Results of T test during general cooperation task in athletes.

| Channel | Corrected_P_Value | T_Value | Cohen_D |
| --- | --- | --- | --- |
| 1 | 0.20 | 1.97 | 0.30 |
| 2 | 0.38 | 1.06 | 0.16 |
| 3 | 0.26 | 1.66 | 0.25 |
| 4 | 0.64 | 0.50 | 0.08 |
| 5 | 0.12 | 2.39 | 0.36 |
| 6 | 0.34 | 1.23 | 0.19 |
| 7 | 0.20 | 1.91 | 0.29 |
| 8 | 0.20 | 1.92 | 0.29 |
| 9 | 0.20 | 1.91 | 0.29 |
| 10 | 0.27 | 1.45 | 0.22 |
| 11 | 0.37 | 1.12 | 0.17 |
| 12 | 0.27 | 1.54 | 0.23 |
| 13 | 0.53 | 0.71 | 0.11 |
| 14 | 0.65 | 0.45 | 0.07 |
| 15 | 0.27 | 1.44 | 0.22 |
| 16 | 0.56 | 0.64 | 0.10 |
| 17 | 0.53 | 0.72 | 0.11 |
| 18 | 0.26 | 1.64 | 0.25 |
| 19 | 0.38 | 1.00 | 0.15 |
| 20 | 0.34 | 1.22 | 0.19 |
| 21 | 0.27 | 1.40 | 0.21 |
| 22 | 0.38 | 1.03 | 0.16 |
| 23 | 0.38 | 1.02 | 0.16 |
| 24 | 0.14 | 2.27 | 0.35 |
| 25 | 0.10 | 2.87 | 0.44 |
| 26 | 0.27 | 1.42 | 0.22 |
| 27 | 0.11 | 2.49 | 0.38 |
| 28 | 0.10 | 2.76 | 0.42 |
| 29 | 0.27 | 1.53 | 0.23 |
| 30 | 0.27 | 1.53 | 0.23 |
| 31 | 0.37 | 1.11 | 0.17 |
| 32 | 0.24 | 1.76 | 0.27 |
| 33 | 0.11 | 2.56 | 0.39 |
| **34** | 0.02 * | 3.77 | 0.58 |

Note: “*” denotes p < .05 after FDR correction. Significant channels are bolded.

**3. Results of general cooperation task in amateurs**

In the amateur group, significant IBS increases during the general cooperative task compared to rest were observed in the following channels: CH9 (t = 2.72, *p* = 0.04, *d* = 0.40), CH23 (t = 2.72, *p* = 0.04, *d* = 0.40), CH26 (t = 2.91, *p* = 0.04, *d* = 0.43), CH27 (t = 2.72, *p* = 0.04, *d* = 0.40), CH28 (t = 2.95, *p* = 0.04, *d* = 0.43), CH29 (t = 3.87, *p* = 0.01, *d* = 0.57), CH34 (t = 2.97, *p* = 0.04, *d* = 0.43), see Figure 3 and Table 3.


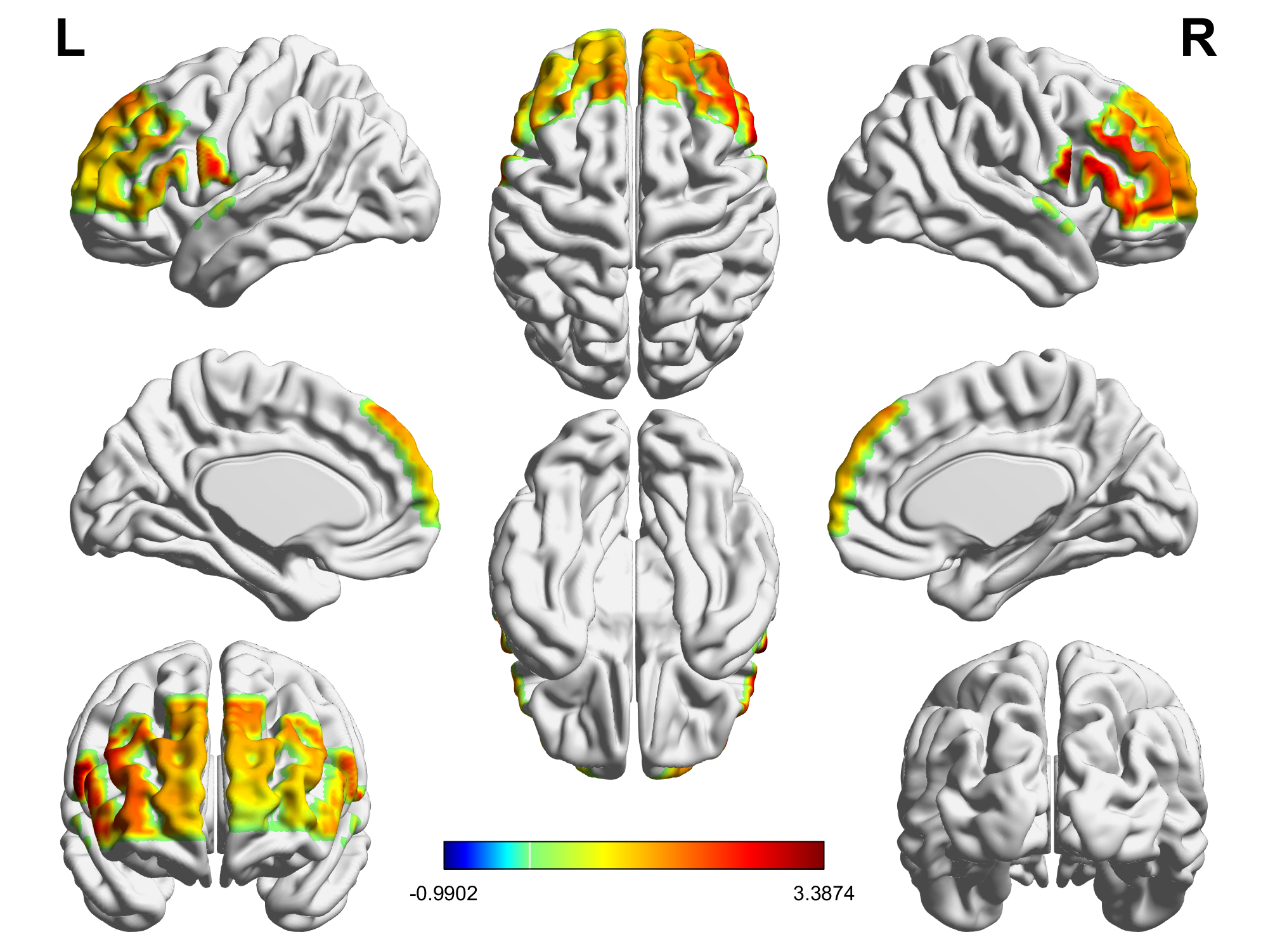


**Figure 3.** T map during general cooperation task in amateurs.

**Table 3.** Results of T test during general cooperation task in amateurs.

| Channel | Corrected_P_Value | T_Value | Cohen_D |
| --- | --- | --- | --- |
| 1 | 0.91 | 0.12 | 0.02 |
| 2 | 0.32 | 1.24 | 0.18 |
| 3 | 0.33 | 1.19 | 0.17 |
| 4 | 0.80 | 0.29 | 0.04 |
| 5 | 0.48 | 0.81 | 0.12 |
| 6 | 0.23 | 1.64 | 0.24 |
| 7 | 0.08 | 2.34 | 0.34 |
| 8 | 0.26 | 1.54 | 0.23 |
| **9** | 0.04 * | 2.72 | 0.40 |
| 10 | 0.08 | 2.29 | 0.33 |
| 11 | 0.37 | 1.07 | 0.16 |
| 12 | 0.40 | 0.98 | 0.14 |
| 13 | 0.33 | 1.18 | 0.17 |
| 14 | 0.26 | 1.45 | 0.21 |
| 15 | 0.58 | 0.61 | 0.09 |
| 16 | 0.36 | 1.10 | 0.16 |
| 17 | 0.07 | 2.45 | 0.36 |
| 18 | 0.26 | 1.52 | 0.22 |
| 19 | 0.41 | 0.94 | 0.14 |
| 20 | 0.29 | 1.34 | 0.20 |
| 21 | 0.53 | 0.71 | 0.10 |
| 22 | 0.10 | 2.12 | 0.31 |
| **23** | 0.04 * | 2.73 | 0.40 |
| 24 | 0.18 | 1.80 | 0.26 |
| 25 | 0.07 | 2.49 | 0.36 |
| **26** | 0.04 * | 2.92 | 0.43 |
| **27** | 0.04 * | 2.72 | 0.40 |
| **28** | 0.04 * | 2.95 | 0.43 |
| **29** | 0.01 * | 3.87 | 0.57 |
| 30 | 0.26 | 1.49 | 0.22 |
| 31 | 0.18 | 1.84 | 0.27 |
| 32 | 0.28 | 1.38 | 0.20 |
| 33 | 0.09 | 2.23 | 0.33 |
| **34** | 0.04 * | 2.97 | 0.43 |

Note: “*” denotes *p* < .05 after FDR correction. Significant channels are bolded in the first column.

**4. ANOVA for each Channel**

To further examine differences between groups and tasks, we extracted IBS values for each experimental condition and conducted a 2 (group: athletes, amateurs) × 2 (task: table tennis, general cooperation) ANOVA for each channel. The results revealed a significant interaction effect (F_(1,89)_ = 4.67, *p* = 0.03, η² = 0.05) at CH17 (F_(1,89)_ = 9.63, *p* = 0.003, η² = 0.10), as the athletes (M = 0.07, SD = 0.09) showed significantly higher IBS than the amateurs (M = 0.01, SD = 0.08) during the table tennis task. In contrast, no significant group difference was found during the general cooperative task (F_(1,89)_ = 1.57, p = 0.21, η² = 0.02). No significant effect was found at CH**8 (*ps* > 0.32), CH14 (*ps* > 0.15) and CH15 (*ps* > 0.64).**

Overall, the channels that showed significant IBS enhancement in the athlete group during the table tennis task did not exhibit comparable increases in either the amateurs or during the general cooperation task. This pattern suggests that the observed IBS increases are not merely a reflection of general cooperative abilities, but are likely associated with the specific task demands of the table tennis and the professional training of athletes. Thus, the enhancement of IBS during the table tennis task is both experience-dependent (differentiating athletes from amateurs) and cognitively specific (linked to joint anticipation rather than general cooperation).
